# Comparative study between fructose 1-6 bisphosphate and histidine-tryptophan-ketoglutarate in liver preservation in rats submitted to total cold ischemia^1^

**DOI:** 10.1590/s0102-865020200060000003

**Published:** 2020-07-08

**Authors:** Fernanda Bombonato Smecellato, Lucas Ricardo Benfatti Marsilli, Julia Eico Nakamura, Maria Cecília Jordani, Paulo Roberto Barbosa Évora, Orlando Castro-e-Silva

**Affiliations:** I Graduate student, Faculdade de Medicina de Marília (FAMEMA), Brazil. Technical procedures; acquisition, analysis and interpretation of data; manuscript preparation.; II Master, Biochemistry, Division of Digestive Surgery, Department of Surgery and Anatomy, Faculdade de Medicina de Ribeirão Preto, Universidade de São Paulo (FMRP-USP), Ribeirao Preto-SP, Brazil. Acquisition and interpretation of data, statistical analysis.; III PhD, Full Professor, Division of Thoracic and Cardiovascular Surgery, Department of Surgery and Anatomy, FMRP-USP, Ribeirao Preto-SP, Brazil. Conception and design of the study, manuscript writing, critical revision.; IV PhD, Full Professor, Department of Surgery and Anatomy, FMRP-USP, Ribeirao Preto-SP, Brazil. Conception and design of the study, analysis and interpretation of data, manuscript writing, critical revision.

**Keywords:** Cold Ischemia, Organ Preservation, Liver Transplantation, Rats

## Abstract

**Purpose:**

To compare Fructose-1,6-Bisphosphate (FBP) to Histidine-Tryptophan-Ketoglutarate (HTK) in liver preservation at cold ischemia.

**Methods:**

Male rats (Sprague-Dawley: 280-340g) divided into three groups (n=7): Control; Fructose-1,6-bisphosphate (FBP); Histidine-Tryptophan-Ketoglutarate (HTK). Animals underwent laparotomy-thoracotomy for perfusion of livers with saline. Livers were removed and deposited into solutions. Mitochondria were isolated to determine State 3 (S3), State 4 (S4), Respiratory Control Ratio (RCR) and Swelling (S). Liver enzymes (AST, ALT, LDH) were determined in solution. At tissue, Malondialdehyde (MDA) and Nitrate (NOx) were determined. All parameters were analyzed at 0.6 and 24 hours of hypothermic preservation. Statistics analysis were made by Mann-Whitney test (p<0.05).

**Results:**

Regarding ALT, there was a difference between FBP-6h/HTK-6h, lower in HTK. Regarding AST, there was a significant difference between FBP-24h/HTK-24h, lower in FBP. Regarding NOx, there was a difference between 0h and 6h, as well as 0h and 24h for both solutions. Regarding S3, there was a significant difference in 24h compared to Control-0h for both solutions, and a significant difference between FBP-6h/FBP-24h. Regarding S4, there was a difference between Control-0h/HTK-24h and FBP-24h/HTK-24h, higher in HTK. There was a difference between Control-0h/FBP-24h for Swelling, higher in FBP.

**Conclusion:**

Fructose-1,6-Bisphosphate showed better performance at nitrate and aspartate aminotransferase compared to histidine-tryptophan-ketoglutarate.

## Introduction

Liver transplantation is used in situations in which patients with liver disease have a life expectancy of less than 20% at the end of 12 months, if they are not transplanted, and in those in which the risk of mortality exceeds the rate resulting from the transplant itself. It is considered a form of treatment for patients with terminal liver failure due to chronic cholestatic and hepatocellular liver diseases, metabolic and vascular liver diseases, primary liver tumor and trauma^1,2^. More than 10.000 liver transplants are performed worldwide per year^3^. This high number is certainly a reflection of innumerable research carried out with the aim of improving the conditions of the transplant. Among these researches, are those involved with the improvement of preservation solutions for liver. With this, it is possible to transport organs over great distances and, consequently, use the vast majority of organs available for donation^1^.

Preservation solutions are important in preservation of tissue damage, avoiding cellular edema, tissue acidosis, free-radical injuries and energy depletion, resulting effects from ischemia-reperfusion injury^4^. Among the solutions available for use, there is the one from the University of Wisconsin (UW), considered the gold standard in liver preservation, although it has some limitations related to risks of biliary complications, high viscosity and high cost. Histidine-Tryptophan-Ketoglutarate (HTK) solution, or Custodiol, is an alternative that has been used in relation to UW, since it has a biliary protective factor, lower viscosity and reduced cost^5^.

The potential of Fructose 1-6 Bisphosphate (FBP) as a preservation solution is still poorly understood, especially when compared to HTK. Thus, this study aims to analyze the conditions of livers of Sprague-Dawley rats preserved in these two solutions (UW, HTK based on studies related to oxidative phosphorylation through parameters involved in mitochondrial respiration and the permeability of the mitochondrial membrane (osmotic swelling), in addition to determinations of reactive species of oxygen (malondialdehyde - MDA) and nitric oxide (NOx) tissue. And liver tissue integrity studies, through determinations of transaminases alanine aminotranferase (ALT) and aspartate aminotransferase (AST) and lactate dehydrogenase (LDH) levels in solutions.

## Methods

The Animal Experimentation Ethics Committee approved the study of the Faculdade de Medicina de Ribeirão Preto of Universidade de São Paulo (FMRP-USP). The rats were kept under controlled light conditions (12 hours of light and 12 hours of darkness), temperature 23ºC, relative humidity 55%, and free access to water and food for rodents.

Male rats of the Sprague-Dawley lineage, weighing between 280-340 g, were divided into three groups (n = 7 per group): 1) Control Group: liver submitted to perfusion with saline immediately before the tests. 2) Fructose 1,6-bisphosphate group (FBP): liver subjected to infusion with saline and then removed and placed in 10 mM FBP solution. 3) Histidine-Tryptophan-Ketoglutarate (HTK) group liver subjected to perfusion with saline and then removed and placed in commercial HTK solution. All parameters were analyzed at 0, 6, and 24 hours of preservation in the media stocked on ice^6^.

### *Operative technique*

The animals were submitted to general anesthesia. The drugs used were: xylazine hydrochloride (20 mg/ml) and ketamine hydrochloride (50 mg/ml). The dosage was 0.15 ml/100g of weight intramuscularly. The anesthetized animal was placed supine on wooden support with the limbs fixed in extension. All groups underwent bilateral subcostal laparotomy followed by bilateral thoracotomy for the perfusion of the liver with chilled saline. The perfusion was performed using hydrostatic pressure after puncturing the right chambers of the heart with a 22 gauge needle catheter connected to a 130 cm high liquid column containing 250mL of the perfusion solution. Immediately after the procedure, the livers were removed and deposited in beakers providing preservation solutions kept at 4ºC. The animals were sacrificed by exsanguination.

### *Preparation of mitochondria*

Isolation of liver mitochondria was performed by differential centrifugation at 4°C. After the surgical procedure. The liver was immediately removed and placed in physiological saline in which it was washed. Then, it was put in medium containing 250 mM sucrose, 1 mM EGTA and 10 mM Hepes-KOH, pH 7.2 in which it was perforated and homogenized in Potter-Elvehjem through 3 cycles of 15 seconds with a 1-minute interval. The homogenate was centrifuged at 770g for 5 minutes, and the resulting supernatant centrifuged at 9800g for 10 minutes. The obtained pellet was suspended in 10 ml of medium containing 250 mM sucrose, 0.3 mM EGTA and 10 mM Hepes-KOH, pH 7.2, and centrifuged at 4500 g for 15 minutes. The final pellet containing the isolated mitochondria was suspended in 0.5 mL of medium containing 250 mM sucrose and 10 mM Hepes-KOH, pH 7.2^7^.

### *Protein determination*

Mitochondrial proteins were determined by the Coomassie Assay (Coomassie plus - Bradford Assay™ kit) at 595nm on a Versamax (Molecular Devices) microplate reader. The results obtained were expressed in mg / mL, using bovine serum albumin as a standard^8^.

### *Oxygen consumption by mitochondria*

Mitochondrial respiration was monitored on a Hansatech-Oxygraph Plus oxygraph equipped with an oxygen electrode in medium containing 125 mM sucrose, 65 mM KCl, 1 mM MgCl_2_, 2 mM KH_2_PO_4_, 0.1 mM EGTA and Hepes 10 mM KOH, pH 7.4, energized with 5 mM potassium succinate. The mitochondrial parameters evaluated were the rate of oxygen consumption in state 4 (basal respiration), the rate of oxygen consumption in state 3 (respiration activated by adenosine diphosphate - ADP) and respiratory control ratio (RCR), which indicates the degree of coupling between oxygen uptake and ADP phosphorylation^9^.

### *Determination of mitochondrial osmotic swelling*

The transition of the internal mitochondrial membrane permeability induced by 20 μM CaCl2 and 1 mM KH2PO4 was determined spectrophotometrically at 540 nm using a Beckman DU 640B spectrophotometer by decreasing the optical density (ΔDO)^10^.

### *Determination of MDA*

At liver homogenates, the colorimetric determination of MDA by its reaction with thiobarbituric acid was performed at 532 nm on a Versamax (Molecular Devices) microplate reader using 1,1,3,3-tetramethoxypropane (0 to 100 μM) as standard and the results obtained were expressed in μM / mg of protein^11^.

### *Determination of NOx*

Collected liver samples were placed in physiological saline at 4°C and stored at -70ºC. The tissue was fragmented and homogenized at 10% (w/v) in 20 mM Tris HCl, pH 7.4. The homogenate was centrifuged at 5000 rpm for 10 minutes, and protein was determined in the supernatant by the Coomassie Plus method (The better Bradford Assay TM Kit – Thermo scientific). The samples were deproteinated by incubation with 95% ethanol for 30 minutes and centrifuged at 10,000 rpm for 5 minutes. The supernatant obtained was used for nitric oxide (NOx) determination by NOx/ozone chemoluminescence14 using 15 µL of the sample. The sample was injected into a reaction chamber containing a reducing agent (0.8% vanadium chloride in 1N HCl) at 95°C that converts nitrate to NOx in equimolar quantities. Using nitrogen gas, NOx is dragged to the chemiluminescence chamber of a Sievers NO Analyzer (Sievers 280i NOA, Sievers, Boulder, CO, USA). Its reaction with ozone detects NO, emitting red light (NOx + O_3_→ NO_2_ + O_2_; NO_2_ → NO_2_ + hv).

The photon emitted by the reaction is detected and converted to an electric signal. The generated current is converted with a digital-to-analog converter and analyzed with a computer. The area under the curve generated by the electric current corresponds to the NOx concentration of the sample. NOx concentration was calculated utilizing a curve using 100 to 1µM sodium nitrate as standard. The results obtained are expressed as µM/mg protein^12^.

### *Determination of ALT, AST, and LDH*

ALT, AST, and LDH were determined on the solution by the kinetic method at 340 nm with the aid of the CELM SB-190 apparatus using a Labtest kit^13^.

### *Statistical analysis*

The results were statistically analyzed by the non-parametric Mann-Whitney test with a significance level of 5% (P <0.05) between the groups. Statistical analyses were performed with GraphPad Prism 6.02 software (GraphPad Software Inc, California).

## Results

**Figure 1:**
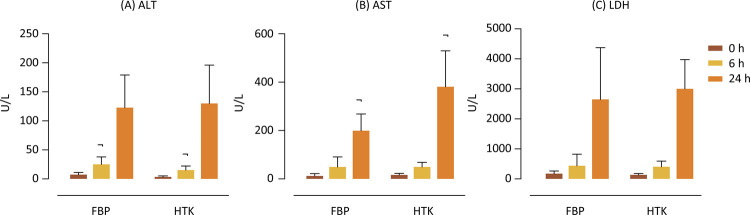
Determination of alanine aminotransferase (ALT) (A), aspartate aminotransferase (AST) (B) and lactate dehydrogenase (LDH) (C) on solution. Values expressed in U/L. p<0.05. ¬Statistical difference between solutions at same time.

The results obtained in the serum determinations of ALT, AST, and LDH are shown in Figure 1 A-C, respectively. The two solutions showed significant differences between studied times (0, 6, and 24) for ALT, AST, and LDH, with increased levels over time. Regarding ALT, there was a difference between the FBP 6h x HTK 6h groups, with lower rates of the enzyme for HTK at that time. Regarding AST, there was a significant difference between the FBP 24h x HTK 24 groups, with much lower levels of the enzyme for FBP, showing a beneficial effect of FBP about HTK at that time. Regarding LDH, there were similar results for both solutions, with no significant differences between them at the same time and shows the same ability of FBP concerning HTK in preserving the liver for this enzyme.

**Figure 2:**
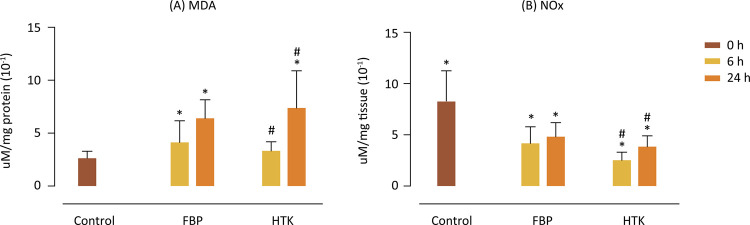
Determination of reactive species with thiobarbituric acid. Values of malondialdehyde (MDA) (A) expressed as μM/mg protein (10-1) and nitric oxide (NOx) (B) expressed as μM/mg tissue (10-2). p<0.05. *Statistical difference between solutions and control group; #Statistical difference between time inside solution.

The results related to oxidative stress, obtained by determining the MDA Figure 2A showed a significant increase in MDA levels with the increase in cold ischemia time, evidenced by the differences between the Control 0h x FBP 6h, Control 0h x groups FBP 24h, Control 0h x HTK 24h. However, there was no difference between the Control 0h x HTK 6h groups, showing protection from lipoperoxidation by HTK in 6 hours, which did not occur with the FBP solution at that time. In 24h, the two solutions showed similar behaviors, since there was no difference between the FBP 24h x HTK 24h groups.

Regarding the NOx Figure 2B, for the two solutions, there was a significant difference between 0 and 6 hours, as well as between 0 and 24 hours, showing a decrease in NOx in the first 6 hours of cold ischemia, which was maintained in 24 hours, although a small difference between HTK 6h x HTK 24h.

**Figure 3:**
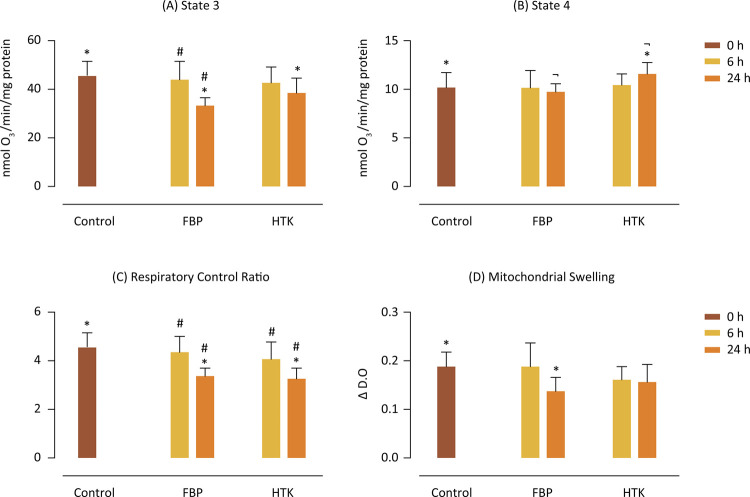
Parameters and mitochondrial processes. (A) Velocity of oxygen consumption by mitochondria in the presence of ADP (state 3). (B) Velocity of oxygen consumption by the mitochondria in the basal state (state 4). Values are expressed as nmol O2/ min/mg protein. (C) Values of the ratio of oxygen consumption velocity by mitochondria at state 3 and state 4 (respiratory control ratio - RCR). (D) Mitochondrial osmotic swelling, induced by CaCl2 and KH2PO4 at 540 nm. Values are expressed as Δ DO (the difference between the initial optical density, before induction, and final, after total induction). p<0.05. *Statistical difference between solutions and control group; #Statistical difference between time inside solution; ¬Statistical difference between solutions at same time.

Regarding the mitochondrial parameters related to oxidative phosphorylation, the oxygen consumption speed in the ADP-activated state (state 3) Figure 3A showed a significant difference only in 24h of cold ischemia compared to the control in time 0h for both solutions, demonstrated by the considerable differences between the Control 0h x FBP 24h and Control 0h x HTK 24h groups. There was also a significant difference between the FBP 6h x FBP 24h groups, showing inhibition of oxygen consumption in 24h compared to 6h for FBP solution, which was not observed between HTK 6h x HTK 24h, which remained similar. As for the speed of oxygen consumption at baseline (state 4), there was a small significant difference between the Control 0h x HTK 24h and FBP 24h x HTK 24h groups, shown in Figure 3B. The ratio between the speed of oxygen consumption between state 3 and state 4 of mitochondrial respiration is called the respiratory control ratio (RCR). It is an important parameter to assess the degree of coupling between oxygen consumption and ATP synthesis by mitochondria. The values are shown in Figure 3C and show significant differences between the control in time 0 and 24h for both solutions. Also for the solutions between them at 6h and 24h, indicating that the worsening in the capacity of ATP synthesis by mitochondria occurs in 24h after cold ischemia and is similar for both solutions. The study of the permeability of the internal mitochondrial membrane was carried out by measuring the optical density at 540 nm, by inducing the mitochondrial osmotic swelling by CaCl_2_ and KH_2_PO_4_. The values presented (Δ DO) were expressed by the difference between the final and initial optical density (before induction), and are shown in Figure 3D. There was a significant difference only between the Control 0h x 24h FBP groups, indicating that mitochondrial edema was only relevant in 24h in the FBP solution.

## Discussion

Some authors suggest that FBP crosses cell membranes to restore depressed glycolytic activity, not only as a metabolic regulator, but also as a metabolic substrate for glycolysis while maintaining intracellular ATP^14^. This can be proven using carbon 13 nuclear magnetic resonance, such that the FBP marked with exogenous carbon 13 can visibly cross the cell membrane^14-17^. It is known that changes in cell membranes during the ischemia process lead to the flow ionic and consequent increase in intracellular Ca^2^ levels. FBP can interact with the biomembrane, modifying ionic permeability, in order to maintain its stability. Thus, FBP has a Ca^2^ chelating action, inhibiting the increase in intracellular Ca^2^ concentration in Kupffer cells isolated from rats^18^. FBP also has anti-inflammatory action, since it is responsible for inhibiting the proliferation of activated mononuclear cells by mitogen, mainly helper T cells, which are most affected. This is due to the FBP’s ability to inhibit IL-2 expression^19^.

The ATP production capacity by the graft depends on the viability of oxidative phosphorylation of the organ after reperfusion, which is directly proportional to the degree of ischemia / reperfusion injury^20,21^. According to the literature, FBP significantly improves hepatic recovery and ATP restoration rates during reperfusion. During ischemia/reperfusion in control livers, state 4 increased progressively, while FBP suppressed this increase, although it did not affect state 3. Thus, the RCR of livers preserved with FBP was higher compared to control during ischemia/reperfusion^22^. In this study, mitochondrial parameters related to oxidative phosphorylation, show that the inhibition of electron transport through the respiratory chain in state 3, resulting from hypoxia is observed only in 24 hours of cold ischemia and that FBP was able, as well as the HTK, in maintaining values similar to the control in the 6h period, both for O_2_ consumption speed in state 3 and the RCR, thus maintaining the ATP synthesis capacity in that period. Accordingly, mitochondrial edema induced by CaCl_2_ and NaH_2_PO_4_, was protected by FBP and HTK in the same period.

Some authors have demonstrated that FBP suppresses the generation of oxygen free radicals and lipid peroxidation, protecting the liver from injury caused to them when the preservation time is less than 18 hours. These results indicate that FBP can suppress lipid peroxidation chain reactions induced by free radicals^23^. In our results, however, MDA levels have been shown to be increased by 6 hours of cold ischemia in the FBP solution, while HTK was able to protect lipoperoxidation during this same period of ischemia. Still in relation to lipid peroxidation, studies in the heart of rats showed that the level of lipid peroxide increased significantly during reoxygenation and its increase was completely suppressed by the administration of FBP. It was also observed that the conversion of xanthine dehydrogenase to xanthine oxidase (a process considered to be the major source of superoxide anion) in the heart during ischemia was inhibited by FBP^23-25^. Therefore, FBP can block the reaction of lipid peroxidation that is induced by ROSs during reperfusion, showing a FBP protective effect against lipid peroxidation induced during reperfusion^18,19^.

Nitric oxide has a dualistic effect on the liver, being cytotoxic or cytoprotective. Among the cytoprotective effects is its role as an inhibitor of the production of free radicals, blocking the release of PGE2 and PGF2, inhibiting platelet aggregation and neutrophil activation, in addition to its vasodilating action, promoting adequate perfusion. Its cytotoxic effects would be in its property of reacting with superoxides to form extremely reactive nitrogenous superoxides and increased lipid peroxidation, among other factors^6,24,26^. Although NO has a dualistic effect on ischemia and reperfusion, experimentally there is evidence to prove the prevalence of the protective effect on liver microcirculation, attenuating the effects of tissue damage. Experimental studies have shown greater post-ischemic cell injury in animals with eNOS deficiency^26^. In addition, researchers have shown that overexpression of eNOS protected rats against liver injury due to ischemia-reperfusion. In addition, there are experimental therapies using the pharmacological induction of the production of endogenous nitrate and administration of exogenous sources of nitrate^23,26,27^. In the present study, the hepatic nitrate determination showed lower values in the ischemic groups at times studied for both solutions, with slightly higher values for FBP. The imbalance can explain the reduced levels of NO between the production of NO by NOS associated with the action of endothelin-1, involved in the ischemia-reperfusion process. Studies of the effect of endothelin-1 in rat livers show that the vasoactive peptide endothelin-1 causes an increase in portal vein pressure leading to severe hepatic vasoconstriction^28^.

The values of the serum determinations of the enzymes ALT, AST, and LDH show an increase in liver damage in the prolongation of ischemia, with better performance for HTK concerning ALT in 6 hours and evident protection for FBP about AST in 24 hours. of cold ischemia.

### *Study limitation*

The reperfusion study in liver transplantation in rats was not performed due to the lack of human resources, both in the research team and in Brazil. The technical difficulty in completing the graft anastomosis in the recipient rat since the procedure is microsurgical and requires people who are appropriately trained to do so. Therefore, it would be interesting for transplantation research centers in Brazil to invest in training professionals to perform such experimental procedures.

## Conclusions

The present study showed better protective effects of the FBP, at NOx and AST, compared to HTK. These data are suggestive of a protective effect on hepatocyte damage and less free radical production with consequent better endothelial function. Since the FBP presented some similar and other superior results, in addition to the economic advantage when compared to the high cost of HTK, these results indicate FBP as a viable solution in the preservation of the liver graft in cold ischemia.

## Footnotes

1 Research performed at Liver Transplantation Unit, Department of Surgery and Anatomy, Faculdade de Medicina de Ribeirão Preto, Universidade de São Paulo (FMRP-USP), and Department of Gastroenterology, Medical School, USP, Sao Paulo-SP, Brazil.
